# Falciparum malaria selected while HIV-1 slaughtered

**DOI:** 10.4103/0971-6866.44109

**Published:** 2008

**Authors:** Kanjaksha Ghosh, Shrimati Shetty, Leenam Mota

**Affiliations:** Department of Haemostasis, National Institute of Immunohaematology (ICMR), 13^th^ Floor, KEM Hospital, Parel, Mumbai, India

Sir,

Human leukocyte antigen (HLA) is one of the most polymorphic systems known to man and its direct involvement in immune response is well known. It is generally accepted that two major infections, i.e. falciparum malaria and tuberculosis, exerted extreme selective pressure to fashion our genome as we see it today. Of the two, falciparum malaria is more important in tropical and sub tropical areas where the disease has been endemic for centuries.

The development of HLA polymorphism and resistance to malaria make interesting reading.[1,2] In different populations, different HLA polymorphisms were linked to resistance to malaria. However HLA B35 came up as one of the alleles in several population groups where falciparum malaria is endemic.[3,4] In India, HLA B35 is a common allele with 12-36% frequency among various populations and caste groups exposed for centuries to falciparum malaria. A study also has shown that HLA B35 can present HIV1 Gag protein (aa20-50 RPGGKKRYMIKHLVWASRELERFALNPGL) to generate cytotoxic T lymphocytes.[5] Simultaneously several studies from all over the world have shown that HLA B35 is associated with faster disease progression in HIV1 infection.[6]

Falciparum malaria, by way of positive selection, has given human beings an Achilles heel in the form of HLA B35 through which the HIV arrow is now passing. The study of HLA in human population gives us an unusual insight i.e. malaria selected HLA B35 positive population, only for it to be slaughtered by HIV1 infection in future. Nature is blind and unforgiving in driving evolution!
